# Association between Anemia and New-Onset Atrial Fibrillation in Critically Ill Patients in the Intensive Care Unit: A Retrospective Cohort Analysis

**DOI:** 10.3390/clinpract12040057

**Published:** 2022-07-12

**Authors:** Gokhan Sertcakacilar, Gunes Ozlem Yildiz

**Affiliations:** 1 Department of Outcomes Research, Anesthesiology Institute, Cleveland Clinic, Cleveland, OH 44195, USA; 2 Department of Anesthesiology and Reanimation, University of Health Science, Bakırköy Dr. Sadi Konuk Education and Research Hospital, 34147 Istanbul, Turkey; drgunesim@hotmail.com

**Keywords:** anemia, atrial fibrillation, intensive care unit, critically ill patients, red blood cell, blood transfusion

## Abstract

New-onset atrial fibrillation (NOAF) is one of the leading causes of morbidity and mortality, especially in older patients in the intensive care unit (ICU). Although many comorbidities are associated with NOAF, the effect of anemia on the onset of atrial fibrillation is still unknown. This study aimed to test the hypothesis that anemia is associated with an increased risk of developing NOAF in critically ill patients in intensive care. We performed a retrospective analysis of critically ill patients who underwent routine hemoglobin and electrocardiography monitoring in the ICU. Receiver operating characteristics analysis determined the hemoglobin (Hb) value that triggered NOAF formation. Bivariate correlation was used to determine the relationship between anemia and NOAF. The incidence of NOAF was 9.9% in the total population, and 12.8% in the patient group with anemia. Analysis of 1931 patients revealed a negative association between anemia and the development of NOAF in the ICU. The stimulatory Hb cut-off value for the formation of NOAF was determined as 9.64 g/dL. Anemia is associated with the development of NOAF in critically ill patients in intensive care.

## 1. Introduction

Tachyarrhythmias are the most common arrhythmia type observed in patients in intensive care, and they cause increased mortality and morbidity. It has been reported that the incidence of atrial or ventricular arrhythmias in general intensive care patients is around 78% [1]. In critically ill patients, new-onset atrial fibrillation (NOAF) is the most common arrhythmia [2,3]. NOAF can accelerate acute heart failure, cause thromboembolic complications, and increase hospital and intensive care unit (ICU) length of stay and mortality [4,5].

It is thought that the development of NOAF in critically ill patients hospitalized in the ICU is triggered by the formation of an arrhythmogenic atrial substrate and initiates arrhythmia [3,6]. NOAF can initially be stimulated by many factors that impair normal electrical conduction, such as hypokalemia, hypomagnesemia, hypovolemia, and parasympathetic and sympathetic activity changes. This may cause atrial foci to develop abnormal automaticity, self-sustaining action potentials, or re-entry circuits [7,8]. Anemia, especially acute anemia, produces intense adrenergic activation, and the adrenergic response due to anemia may trigger the development of NOAF in predisposed patients. Another mechanism that predisposes anemia to develop NOAF is ischemic damage to atrial myocytes and myocardial conduction cells [9].

Anemia is a well-known complication of critical illness. Almost all patients staying in the ICU for more than seven days experience anemia, and more than 75% of critically ill survivors are anemic at hospital discharge [10,11]. The World Health Organization (WHO) defines anemia as hemoglobin concentrations of less than 13 g dL^−1^ in men and less than 12 g dL^−1^ in women. This definition is valid for general public health, but different threshold values may be appropriate for patients in the ICU [12]. The prevalence of anemia, as with NOAF, is age-dependent and increases as the population ages. The incidence of the development of NOAF reaches approximately 13% in patients over 75 years of age [13], and the prevalence of anemia is around 11% and 10.2% in men and women over 65 years, respectively. It increases up to 20–25% after the age of 85 years [14]. There is little evidence of an association between anemia and the incidence of the development of NOAF, although it is widespread in patients hospitalized in the ICU. This study aimed to determine whether there was a correlation between NOAF and anemia in older patients hospitalized in the ICU.

## 2. Materials and Methods

### 2.1. Study Design and Patient Selection

All patients were enrolled who received intensive care treatment in a single center between 1 January 2012 and 30 November 2021. They were obtained using structured query language queries from the EMRall-QlinICUImdSoftMetavision clinical decision support system and evaluated retrospectively. Based on the WHO criteria, the patients were divided into two groups, anemic and non-anemic, according to their hemoglobin (Hb) levels. The inclusion criteria were patients aged over 45 years and treated in the ICU for more than 72 h. In addition, new-onset AF, including paroxysmal AF, defined as rhythm classification by continuous ECG monitoring or 12-lead ECG, was included. Patients at high risk of developing AF, such as those with a known history of AF or under treatment, those admitted to the ICU after thoracic surgery, who had undergone or would undergo pacemaker implantation or surgical ablation, and had missing data were excluded from the study.

Demographic data of the patients, comorbid diseases, diagnosis at hospitalization, laboratory findings, length of stay in the ICU, and mortality data were evaluated. Furthermore, data obtained from the Simplified Acute Physiology Score II (SAPS II), the Acute Physiology and Chronic Health Evaluation II (APACHE II), and the Sequential Organ Failure Assessment (SOFA) scoring systems, which are the most commonly used disease severity determination scores, were included in the study. This study was conducted in accordance with the Declaration of Helsinki (revised in 2013). The study was approved by the ethics committee board of the Bakırköy Dr. Sadi Konuk Training and Research Hospital (No.: 2021-23-12/2021-502), and individual consent for this retrospective analysis was waived.

### 2.2. Red Blood Cell Transfusion Protocol

In line with the standard treatment protocol of the ICU, daily Hb and hematocrit (Hct) values are monitored in critically ill patients during and after hospitalization. All patients are transfused according to a protocol recommended by the National Anesthesiology and Reanimation Association transfusion guidelines [15]. Accordingly, Hb = 8 g/dL (4.96 mmol/L) for patients aged over 60 years and average risk and Hb = 10 g/dL (6.21 mmol/L) for high-risk patients regardless of age were accepted as the transfusion limit. In addition, anemia signs and symptoms such as urine volume below 30 mL/h, tachycardia (>100 beats/minute), and hypotension (mean blood pressure < 60 mm Hg), independent of Hb level, are effective factors in deciding on red blood cell (RBC) transfusion. The intensive care physician decides on allogeneic RBC transfusion in the ICU. The local blood bank provides packed red blood cells with mean values in a buffy coat-removed erythrocyte suspension: volume 200 mL, Hb 15 g/dL, Hct 65–75%, and leukocytes 0.4 × 10^9^/L.

### 2.3. Outcome Measurements

The study’s primary outcome measure was to evaluate whether anemia affected the development of NOAF according to Hb levels in patients treated in the ICU. Secondary outcome measures were the mortality rate in patients with NOAF compared with other patients and the length of stay in the ICU.

### 2.4. Statistical Analysis

The data collected in the study were evaluated using the SPSS 22.00 version of the Windows 10 statistical program. The Kolmogorov–Smirnov test was used to check the normality of data distribution. Numerical variables with a normal distribution were expressed as the mean ± SD, and numerical variables with a skewed distribution were expressed as the median and interquartile ranges (IQRs). For descriptive statistics, categorical variables were given as percentages (%) and numerical variables as mean ± standard deviation. In comparing the quantitative data of the groups when normality conditions were met, the two-sample independent t-test was used, and the Chi-square test was used when the variables were qualitative. The Mann–Whitney U test was used for quantitative variable data comparisons where normality conditions were not met. The statistical significance level of alpha was accepted as *p* < 0.05. Receiver operating characteristics (ROC) analysis was performed between NOAF and Hb values of patients to reveal a cut-off point and sensitivity and specificity percentages, and the area under the curve (AUC) was calculated. A bivariate Spearman’s correlation analysis was used to assess the correlations between the variables. Correlation coefficients measured the strength and direction of the linear relationship between two variables. Those independent predictors derived from logistic regression were selected and incorporated into logistic regression models. A *p* value of <0.05 was considered statistically significant for all tests.

## 3. Results

**Figure 1:**
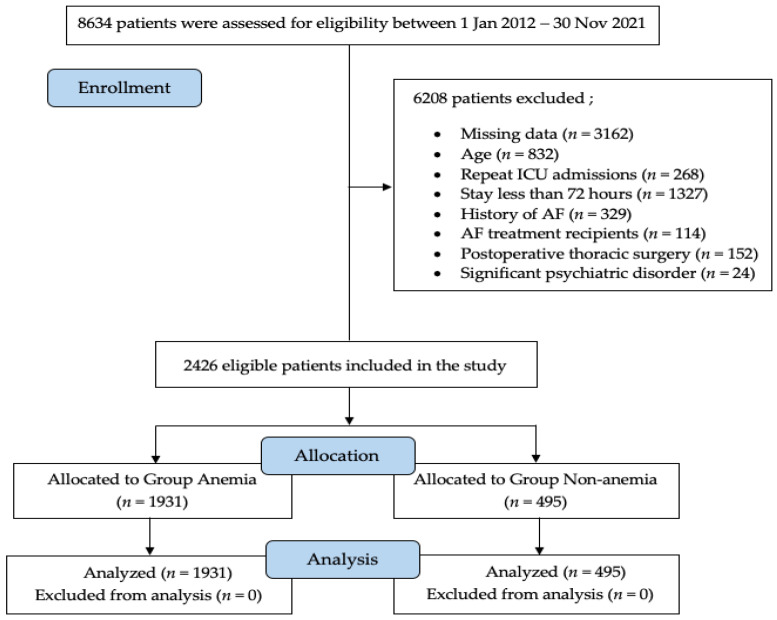
Flow chart of the study. ICU, intensive care unit; AF, atrial fibrillation.

Of the 8634 patients admitted to the ICU during the specified study period, 6208 patients who did not meet the admission criteria were excluded from the study. A total of 2426 patients were analyzed (Figure 1). Slightly over half (54.2%) of the study population was male, and the mean age was 75.2 years. The most common comorbid disease was hypertension (*n* = 902; 36.9%), and the most common admission diagnosis was respiratory failure (*n* = 721; 29.7%). The mean APACHE II score was 19.3 ± 7.4, the SAPS III score was 51.1 ± 13.9, and the SOFA score was 6.43 ± 3.73. Mechanical ventilation was administered to 1756 (72.3%) patients. The mean length of stay in the ICU was found as 148 ± 210.9 h. NOAF developed in 311 (12.8%) patients. The mortality rate in patients with NOAF was 34.4%. One thousand nine hundred thirty-one (79.5%) patients were anemic, and 495 (20.4%) had normal Hb levels. The demographic data of the patients were similar (Table 1). Comorbid diseases such as coronary artery disease, diabetes mellitus, cerebrovascular disease, renal failure, and cancer were statistically significantly higher in anemic patients. The most common admission diagnosis in all groups upon admission to the ICU was respiratory failure (29.7%). In the comparison between the groups, sepsis and renal disease were found to be more common in patients with anemia compared with the patients in the other group (*p* < 0.001 and *p* = 0.004, respectively).

Correspondingly, urea and creatinine, white blood cell (WBC), C-reactive protein (CRP), and procalcitonin values were found to be significantly higher in the patient group with anemia (*p* < 0.001). In addition, the APACHE II, SAPS III, and SOFA scores of the patients in this group were again statistically higher (*p* < 0.001) (Table 2). Similarly, ICU scores were found to be significantly higher in the subgroup of patients who were diagnosed as having AF (*p* = 0.002, *p* < 0.001, and *p* < 0.001) (Table 3). In multivariate analyses, after adjustment for potential confounders in the NOAF group (age, gender, BMI, sepsis, anemia, renal failure, and APACHE II, SOFA, SAPS III), the relationship between anemia and AF onset remained significant (*p* = 0.001, Table 4).

**Figure 2:**
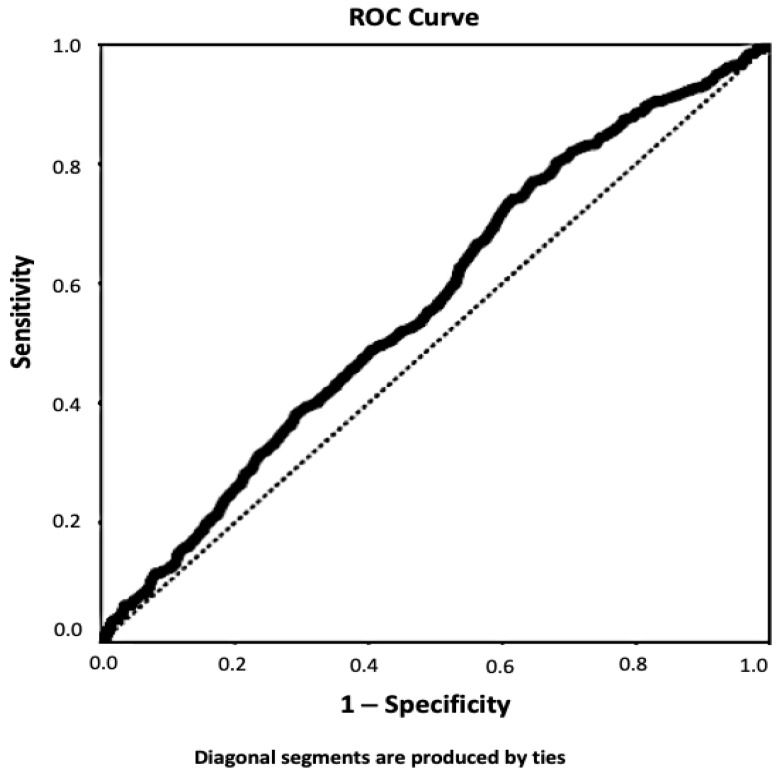
ROC analysis curves of hemoglobin concentration and NOAF according to anemia group. ROC, receiver operating characteristic; NOAF, new-onset atrial fibrillation.

ROC analysis was performed to determine the Hb cut-off value that stimulated the formation of AF in patients hospitalized in the ICU. Accordingly, it was determined that NOAF could develop with 53.3% specificity and 52.7% sensitivity in Hb values below 9.64 g/dL in critically ill patients in the ICU (AUC: 0.566, *p* = 0.001) (Figure 2).

**Figure 3:**
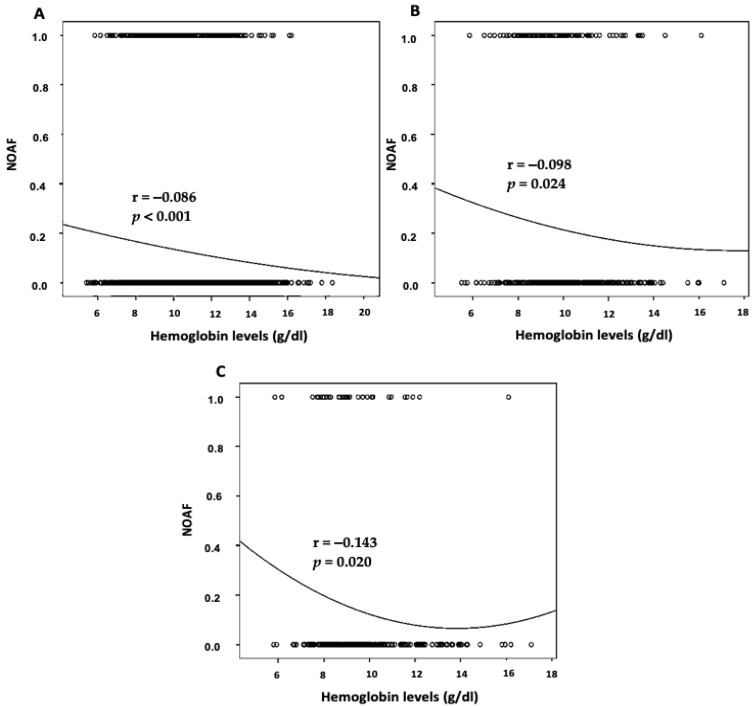
Correlation between serum hemoglobin levels (g/dL) and NOAF in critically ill patients during intensive care unit stay. (**A**) Spearman’s correlation coefficient (r)  = − 0.086, *p* < 0.001 in all patients. (**B**) Spearman’s correlation coefficient (r)  = −0.098, *p* = 0.024 only in patients with sepsis. (**C**) Spearman’s correlation coefficient (r)  = −0.143, *p* =  0.020 only in patients with renal failure (NOAF, new-onset atrial fibrillation).

The bivariate Pearson correlation analysis was performed to study the potential associations of different factors such as sepsis and renal failure with serum hemoglobin levels and NOAF among patients critically ill in the ICU. The analysis revealed that the serum hemoglobin level had a significant negative association with the frequency of NOAF. In addition to this, NOAF was negatively and significantly associated with sepsis and renal failure (*p* < 0.05) (Figure 3).

## 4. Discussion

Our retrospective observational study revealed that NOAF occurred more frequently in the presence of anemia. When combined with existing predisposing factors, the incidence of NOAF increased. Remarkably, the incidence of NOAF development was 9.9% in the patient group without anemia, and 13.6% in 1931 patients with anemia. Our analysis shows that AF is rarer in patients without anemia. After adjusting for potential variables in the NOAF group, the association between anemia and AF remained significant in the multivariate analysis.

The causes of anemia in the analyzed patients mainly were age-related malnutrition problems and anemia of chronic disease. Comorbid diseases such as coronary artery disease, diabetes mellitus, cerebrovascular disease, renal failure, and cancer were statistically significantly higher in anemic patients.

### 4.1. Implications for the Pathophysiology of Anemia and AF

The association between anemia and NOAF in the ICU is remarkable because, unlike other risk factors, including core cardiovascular risk, Hb concentrations are a potentially modifiable factor. Low Hb concentrations, included in the definition of anemia, are a possible marker for the pathophysiologic processes that favor the development of NOAF. However, a causal relationship may also be considered because NOAF is seen to occur due to an insufficient response to myocardial needs in anemia and atrial ischemia [9]. Presumably, hypotension may compromise myocardial perfusion, particularly in patients with anemia. Given that low Hb levels are thought to be causally related to insufficient perfusion, prevention of anemia may prevent the development of NOAF. Based on this, the authors determined that there was a negative correlation between the development of AF and Hb values and that the development of NOAF was more common, especially with Hb values below 9.64 mg/dL. We believe that this cut-off value is a valuable finding for critically ill patients in the ICU.

In the review of the epidemiology of NOAF, criteria such as age, Simplified Acute Physiology Score II (SAPS II), the Acute Physiology and Chronic Health Evaluation II (APACHE II), and systemic inflammatory response syndrome (SIRS) have been emphasized to date [4,16]. In these studies, it was stated that the presence of systemic inflammation in patients in the ICU might have a role in triggering NOAF. The presence of immune cells mediating the inflammatory response in the systemic circulation and subsequent inflammation may stimulate the onset or recurrence of atrial fibrillation [6]. In addition, mediators of the inflammatory response can alter atrial electrophysiologic and structural substrates, thus leading to increased susceptibility to NOAF [17]. In the present study, independent of anemia, CRP, and procalcitonin, indicators of inflammation were found to be statistically higher in the patient group with NOAF. Furthermore, it was observed that the mentioned APACHE II and SAPS III scores and sepsis diagnoses were similarly higher.

However, studies conducted to date have neglected to consider that anemia may be an etiologic factor. In the present study, the incidence of NOAF was statistically higher, especially in older patients with anemia. The results of previous studies and the presented study show that especially newly formed anemia, based on inflammation (anemia of inflammation) or existing anemia that deepens further, may accelerate the development of NOAF in patients in the ICU.

Anemia of inflammation (AI) occurs due to stimulation of hepcidin synthesis in inflammatory diseases, including connective tissue diseases, infections, certain cancers, and chronic kidney disease. As a result of the stimulation of hepcidin by proinflammatory cytokines and, most notably, interleukin (IL)-6 [18,19], anemia occurs because iron is retained in the cells, and its flow into the plasma decreases [20]. A retrospective cohort study found that high IL-6 levels in more than 50% of the patients were associated with clinical outcomes such as iron deficiency, decreased left ventricular ejection fraction, and NOAF [21]. In light of this evidence, we can state that inflammation, as the main reason, contributes to the emergence of anemia or deepens existing anemia. These two leading causes increase the incidence of NOAF.

Furthermore, studies have reported that hypovolemia due to bleeding or another similar reason plays an essential role in the precipitation of atrial fibrillation and the underlying condition. In these patients, conversion to sinus rhythm after transfusion has proven that hypovolemia is a triggering factor that must be corrected. In general, these patients were found to have low or normal pulmonary artery occlusion pressure. There is a known poor absolute correlation between the measured circulating blood volume and the pulmonary artery occlusion pressure [22,23].

### 4.2. Blood Transfusion to Prevent the Development of AF in Patients with Anemia

In general, regardless of its etiology, allogeneic blood transfusion is one of the first options in the treatment of anemia in the ICU. However, blood transfusion is expensive and not an innocuous treatment. Transfusions are associated with pulmonary complications, sepsis, thromboembolic events, and mortality [24,25,26,27]. However, RBC transfusion may be harmful even in the geriatric age group, where anemia is tolerated less, in patients with cardiovascular disease, cancer, or in patients receiving beta-blocker therapy [28,29]. Although current guidelines recommend avoiding RBC transfusions until Hb levels reach 7–8 g/dL in clinically stable patients who have undergone surgery [24,25], critically ill older patients in the ICU should be excluded from this situation. Recent research and systematic reviews have found no evidence that a restrictive transfusion strategy affects 30-day mortality, mortality at other time points, or morbidity (i.e., cardiac events, myocardial infarction, stroke, pneumonia, thromboembolism, infection) compared to a liberal transfusion strategy [30]. The findings provide good evidence that transfusions with allogeneic RBCs can be avoided in most patients with hemoglobin thresholds between 7.0 g/dL and 8.0 g/dL. However, existing studies have reported that some subsets of patients may benefit from RBCs to maintain higher hemoglobin concentrations and that future research should focus on these clinical contexts [31]. A recent study stated that a threshold value of 7 g/dL for RBC transfusion in critically ill patients in the intensive care unit is generally reliable [26]. The authors believe that a cut-off Hb value, determined as a general recommendation, can be decisive in this regard. A contrasting view is that blood transfusions may cause NOAF by causing mediator release [32]. Allogeneic RBC transfusion to correct anemia may mediate the development of NOAF as a predictive factor, which may overshadow the potential benefit of correcting anemia. For this reason, although the authors found a negative correlation between Hb values and the development of NOAF, it is still unclear whether the relationship between anemia and NOAF in patients in the ICU is causal. At the same time, it is unclear whether the correction of anemia with RBC transfusions can change the risk of developing NOAF because there are many morbidity factors, such as sepsis, kidney failure, diabetes, cirrhosis, and cancer, that may contribute to the development of NOAF in critically ill patients hospitalized in the ICU. The relationship between these diseases accompanying the process in the ICU and NOAF has been demonstrated in studies [33,34,35].

### 4.3. Clinical Results of NOAF

In the presented study, there were several significant differences in addition to hemoglobin levels between patients with and without NOAF. They were more severely ill, as indicated by the APACHE II, SOFA, and SAPS III scores. They also had more frequent sepsis, which has been shown to be associated with atrial fibrillation in the ICU. Moreover, patients with anemia had more chronic kidney disease, diabetes, and, most importantly, cardiac disease, which may affect the results of the current study. In the multivariable regression analysis performed to resolve this uncertainty, the association between anemia and NOAF remained significant after adjustment for possible confounding factors. Although there was no difference between the groups in terms of vasoactive agents’ use, it should be considered that vasoactive drugs may contribute to the pathophysiological processes that support the development of NOAF.

In this study, it was determined that the length of stay in the ICU was significantly longer in patients who developed NOAF compared with other patients. Similar studies have reported that NOAF prolongs the length of stay in the ICU and hospital [5,14]. Klouwenberg et al. reported a 3.5-day increase in ICU stay in patients presenting with sepsis and developing NOAF compared with those without AF [36]. The present study also found that the mortality rate in patients who developed NOAF was higher than in other patients. AF is known to be associated with severe morbidity and mortality in adults [32]. It was reported that ICU mortality increased from 37.7% to 56.3% in patients treated for severe sepsis who developed NOAF [37].

### 4.4. Strengths and Limitations of the Study

Although studies to date have generally identified certain risk factors for the formation of NOAF, evaluating the occurrence of NOAF, especially in patients hospitalized in the ICU, may shed more light on the underlying risk factors that lead to severe consequences. The strength of our design was to include patients who were followed for more than 72 h because NOAF usually develops during the process of staying in the intensive care unit. In addition, all patients included in the study were aged 45 years or older and had routine ICU follow-ups. All hemodynamic data were monitored and recorded until they left the ICU. Therefore, the development of NOAF was detected instantly, its treatment was started, and it was recorded. Our study included instant diagnoses and results in patients at risk of NOAF; thus, our cohort should be interpreted accordingly.

Although our study data have strengths, such as minimizing data loss and preventing human errors due to an electronic query by a clinical decision support system, our study has some limitations. First, having a population from a single center prevents the generalization of the results. The retrospective design of the study may entail the influence of confounding factors that could affect the results and the risk of bias. Moreover, due to the retrospective nature of our analysis, the type and quality of data found in medical records were limited. In addition, the population tested was heterogeneous, with some having anemia with chronic disease, whereas others were patients with acute bleeding and needed a transfusion, major surgery, or trauma. Furthermore, the impact of volume substitution and postoperative ventilation settings on AF rates could have impacted our results and acted as non-identified confounders.

## 5. Conclusions

The authors found a strong correlation between anemia and NOAF in patients in the ICU. Whether this relationship is causal has not yet been determined. However, considering that myocardial supply–demand mismatch and atrial ischemia may stimulate NOAF during the ICU, it is possible to correlate the results obtained.

## Figures and Tables

**Table 1:** Patient demographic and medical characteristics during the ICU stay.

	Anemia Group (*n* = 1931)	Non-Anemia Group (*n* = 495)	*p* Value
Age, yr	75.54 ± 6.98	74.95 ± 7.61	0.11
Gender			
Male	1042 (54%)	273 (55.2%)	0.635
Female	889 (46%)	222 (44,8%)	
BMI	27.3 ± 5.53	27.5 ± 5.42	0.363
Comorbidities			
Hypertension	722 (37.4%)	180 (36.4%)	0.673
Cardiac disease	607 (31.4%)	130 (26.3%)	0.026
Diabetes mellitus	442 (22.9%)	82 (16.6%)	0.002
Pulmonary disease	173 (9%)	54 (10.9%)	0.184
Cerebrovascular disease	164 (8.5%)	23 (4.6%)	0.004
CKD	181 (9.4%)	21 (4.2%)	<0.001
Metastatic cancer	228 (11.8%)	41 (8.3%)	0.026
Liver disease	71 (3.7%)	14 (2.8%)	0.360
Other	48 (2.5%)	11 (2.2%)	0.734
Admission diagnosis			
Sepsis	483 (25%)	50 (10.1%)	<0.001
Pulmonary disease	561 (29.1%)	160 (32.3%)	0.155
Cardiac disease	158 (8.2%)	63 (12.7%)	0.002
Cerebrovascular disease	200 (10.4%)	75 (15.2%)	0.003
Postoperative care	446 (23.1%)	173 (34.9%)	<0.001
Trauma	42 (2.2%)	9 (1.8%)	0.621
Renal failure	228 (11.8%)	36 (7.3%)	0.004
Metastatic cancer	179 (9.3%)	79 (16%)	<0.001
Other	136 (7%)	17 (3.4%)	0.003
NOAF diagnosis	262 (13.6%)	49 (9.9%)	0.029
ICU risk scores			
APACHE II	20.03 ± 7.83	18.27 ± 7.02	<0.001
SAPS III	52.69 ± 14.91	49.31 ± 13.04	<0.001
SOFA	6.99 ± 4.02	5.87 ± 3.44	<0.001
Mechanic ventilation	1458 (75.5%)	298 (60.2%)	<0.001
CRRT	578 (29.9%)	147 (29.7%)	0.919
Use of vasoactive agents	1203 (62.3%)	311 (62.8%)	0.743
RBC transfusion (mL)	428.41 ± 276.28	164.25 ± 126.67	<0.001
LOS in ICU (h)	205.26 ± 276.53	90.86 ± 145.33	<0.001
ICU mortality	613 (31.7%)	124 (25.1%)	0.004

Data are presented as mean ± standard deviation (SD) or number (%). BMI, body mass index; CKD, chronic kidney disease; NOAF, new-onset atrial fibrillation; APACHE, Acute Physiology and Chronic Health Evaluation; SAPS, Simplified Acute Physiology; SOFA, Sequential Organ Failure Assessment; CRRT, continued renal replacement therapy; RBC, red blood cell; LOS, length of stay; ICU, intensive care unit.

**Table 2:** Baseline characteristics by average laboratory parameters during the ICU stay.

	Anemia Group (*n* = 1931)	Non-Anemia Group (*n* = 495)	*p* Value
Hemoglobin (g/dL)	9.79 ± 1.43	13.57 ± 1.08	<0.001
Hematocrit (%)	30.61 ± 4.58	41.63 ± 3.79	<0.001
Platelet (×10^9^/L)	228.19 ± 105.64	218.25 ± 84.62	0.094
WBC (×10^9^/L)	14.18 ± 10.14	13.78 ± 6.72	0.940
CRP (mg/L)	84.38 ± 80.85	61.22 ± 78.75	<0.001
Procalcitonin (mcg/L)	7.91 ± 25.75	6.52 ± 15.41	<0.001
Glucose (mg/dL)	151.04 ± 65.71	155.98 ± 62.48	0.086
ALT (U/L)	122.23 ± 335.33	149.70 ± 513.95	0.404
AST (U/L)	256.03 ± 742.51	240.77 ± 782.88	0.006
BUN	85.12 ± 52.26	64.87 ± 42.66	<0.001
Blood creatinine (mg/dL)	1.64 ± 1.29	1.28 ± 0.90	<0.001
Albumin (mg/dL)	18.76 ± 9.20	26.18 ± 6.40	<0.001
Sodium (mmol/L)	138.82 ± 6.13	138.28 ± 10.19	0.475
Potassium (mmol/L)	4.24 ± 0.72	4.31 ± 0.70	0.017
Magnesium (mg/dL)	2.02 ± 0.40	2.07 ± 0.42	0.056
Chlorine (mmol/L)	107.59 ± 5.92	107.69 ± 5.93	0.678
Blood gas analysis			
PH	7.36 ± 1.20	7.33 ± 0.19	0.532
PO_2_ (mmHg)	88.56 ± 38.34	88.04 ± 36.20	0.966
PCO_2_ (mmHg)	43.28 ± 12.36	44.64 ± 12.28	0.010
HCO_3_ (mEq/L)	22.49 ± 4.98	23.66 ± 4.62	<0.001
Lactate (mmol/L)	3.18 ± 3.31	2.72 ± 2.65	0.096
BE	−2.46 ± 6.76	−2.08 ± 7.30	0.007

Data are presented as mean ± standard deviation (SD). WBC, white blood cell; CRP, C-reactive protein; ALT, alanine aminotransferase; AST, aspartate aminotransferase; BUN, blood urea nitrogen; PH, power of hydrogen; PCO_2_, partial pressure of carbon dioxide; PO_2_, partial pressure of oxygen; HCO_3_, bicarbonate; BE, base excess.

**Table 3:** Comparison of the medical and laboratory characteristics of patients diagnosed with NOAF.

	Patients with NOAF (*n* = 311)	Patients without NOAF (*n* = 2115)	*p* Value
Admission diagnosis			
Sepsis	118 (37.9%)	415 (19.6%)	<0.001
Renal failure	37 (11.9%)	227 (10.7%)	0.538
Postoperative care	53 (17%)	566 (26.8%)	<0.001
Metastatic cancer	31 (10%)	227 (10.7%)	0.683
Other diagnosis	128 (41.2%)	850 (40.2%)	0.745
Baseline characteristics			
Hemoglobin (g/dL)	10.10 ± 1.91	10.63 ± 2.05	<0.001
Hematocrit (%)	31.49 ± 5.95	33.06 ± 6.29	<0.001
Platelet (×10^9^/L)	209.85 ± 109.16	228.56 ± 100.44	<0.001
WBC (×10^9^/L)	15.56 ± 18.60	13.89 ± 7.28	0.739
CRP (mg/L)	102.00 ± 90.66	76.37 ± 78.91	<0.001
Procalcitonin (mcg/L)	9.97 ± 20.79	7.28 ± 24.43	<0.001
Mechanic ventilation	234 (75.24%)	1522 (71.96%)	0.227
PEEP (cmH_2_O)	5.1 (4.8–5.6)	5.3 (5.1–5.8)	0.443
Tidal volume	482 (435–528)	476 (427–534)	0.622
Tidal volume (mL/kg)	6.51 (5.86–7.44)	6.49 (5.74–7.21)	0.289
Cardiac ultrasound data			
LAD (mm)	38.3 ± 5.8	40.1 ± 6.4	0.339
LVEDD (mm)	46.5 ± 5.4	45.9 ± 6.1	0.069
LVEDV (mL)	55.8 ± 7.2	58.6 ± 9.8	0.075
LVEF (%)	51.4 ± 12.0	52.8 ± 11.2	0.042
ICU risk scores			
APACHE II	20.87 ± 7.81	19.49 ± 7.68	0.002
SOFA	7.72 ± 3.88	6.62 ± 3.93	<0.001
SAPS III	55.12 ± 15.64	51.54 ± 14.39	<0.001
Use of vasoactive agents	192 (61.8%)	1315 (62.2%)	0.639
RBC transfusion (mL)	386.40 ± 285.12	378.32 ± 290.26	0.259
LOS in ICU (h)	202.96 ± 231.00	178.82 ± 263.23	<0.001
ICU mortality	107 (34.4%)	610 (28.8%)	0.045

Data are presented as mean ± standard deviation (SD), median (IQR) or number (%). WBC, white blood cell; CRP, C-reactive protein; PEEP, positive end-expiratory pressure; LAD, left atrial diameter; LVEDD, left ventricular end-diastolic diameter, LVEDV, left ventricular end-diastolic volume; LVEF, left ventricular ejection fraction; APACHE, Acute Physiology and Chronic Health Evaluation; SAPS, Simplifed Acute Physiology; SOFA, Sequential Organ Failure Assessment; RBC, red blood cell; LOS, length of stay; ICU, intensive care unit.

**Table 4:** Univariate and multiple variation analysis of anemia in NOAF group.

Covariations	*p* Value	OR	95% CI	*p* adj	OR adj	95% CI adj
Age	0.772	1.002	0.986–1.019	0.971	1.000	0.983–1.018
Gender	<0.001	0.518	0.402–0.666	0.142	0.779	0.557–1.087
BMI	0.082	0.979	0.957–1.003	0.834	0.997	0.973–1.022
Sepsis	<0.001	2.463	1.912–3.173	0.151	1.457	0.872–2.436
Anemia	0.030	1.429	1.035–1.973	0.001	2.865	1.511–5.197
Pulmonary disease	<0.001	2.263	1.776–2.885	0.280	1.309	0.803–2.133
Cardiac disease	<0.001	1.986	1.404–2.809	0.388	0.783	0.450–1.364
Renal failure	<0.001	2.358	1.723–3.227	0.258	1.378	0.790–2.405
APACHE II	0.003	1.023	1.008–1.038	0.319	0.988	0.966–1.012
SAPS III	0.000	1.016	1.008–1.023	0.176	1.008	0.996–1.021
SOFA	<0.001	1.070	1.039–1.101	0.034	1.049	1.004–1.097

OR, odds ratio; BMI, body mass index; APACHE, Acute Physiology and Chronic Health Evaluation; SAPS, Simplified Acute Physiology; SOFA, Sequential Organ Failure Assessment.

## Data Availability

The data presented in this study are available on request from the corresponding author. The data are not publicly available due to privacy reasons.
